# Effect of Water-Based Disinfectants or Air-Drying on Dimensional Changes in a Thermoplastic Orthodontic Aligner

**DOI:** 10.3390/ma14247850

**Published:** 2021-12-18

**Authors:** Davide Bresolato, Andrea Volpato, Lorenzo Favero, Riccardo Favero

**Affiliations:** Orthodontic Unit, Dental Clinic, Department of Neurosciences, University of Padova, 35128 Padova, Italy; davide.bresolato@gmail.com (D.B.); lorenzo.favero@unipd.it (L.F.); rickyfavero@msn.com (R.F.)

**Keywords:** aligners, PET-G, orthodontics

## Abstract

The polymer structure of thermoplastic materials currently used to make aligners is altered by the oral conditions and this negatively affects their capacity to move teeth. This study aimed to compare different options for storing aligners when not in use by superimposing successive 3D images to identify which storage method least affects material shape and weight. Fifty PET-G aligners, produced using the CA Digital method, were divided into four groups (1A, 1B, 1C, and 2D) and were stored for 18 h a day in artificial saliva at 37 °C. Then, to mimic their storage conditions when not in use, aligners in group 1A were immersed for the remaining 8 hours a day in bicarbonate solution, those in group 1B in chlorhexidine solution, those in group 1C in distilled water, and those in group 2D were stored dry. The samples were scanned at the baseline (before the immersion cycles began) and again two weeks later. The digital scans were superimposed and the median deformation, its variability, and weight differences were recorded for each group. Statistical analysis showed aligner deformation (expansion) in all three groups stored in wet conditions, with a statistically significant difference between groups 1A and 1C. Aligners in group 2D shrank slightly, and to a significantly greater degree with respect to group 1C. Variability in the degree of deformation was similar among the three groups stored in wet conditions, but significantly greater in group 2D. Weight gains were recorded in all four groups, the smallest in group 2D and the largest in group 1A. Storing aligners in dry conditions promoted lower deformation in the material, involving a slight shrinkage, whereas wet storage conditions caused an expansion of the aligner, especially when distilled water is used.

## 1. Introduction

Clear aligners, a relatively new entry in orthodontics, have aroused considerable clinical interest because of their appealing invisibility and limited invasiveness. The demand for orthodontic treatment using aligners has seen exponential growth in the last 15 years [1], associated with broader clinical indications for their use [2] and continuing advances in the methods and materials used to fashion orthodontic appliances. Since aligners were first introduced in the 1940s [3], numerous different materials have been proposed and employed with a view to achieving the planned biomechanical results. An ideal aligner material should have the possess following features: high spring back, low stiffness, good formability, and high “stored energy,” biocompatibility, and ambient temperature stability.

The mechanical characteristics of an aligner correlate closely with the efficacy of orthodontic force exertion [4]. However, none of the currently available materials fully satisfy all the above-listed requirements simultaneously [5]. The obvious drawbacks of the first generation of aligners related (among other things) to inadequacies of the materials available at the time: acetate, vinyl, polyethylene and butyrate [6] were all lacking in some respect. Even the arrival of cellulose butyrate, polyurethane, and polycarbonate in the mid-1970s [7] failed to achieve significant improvements in the clinical results. An interesting variant proposed by Sheridan [8] in the 1990s involved using copolyester sheets 0.030” thick to obtain aligners that transmitted various information to the tooth through the orthodontic force they exerted.

The real revolution in aligner fabrication came in the early years of the new century, when the whole process was industrialized and gradually perfected thanks both to CAD/CAM digital technologies (which could ensure precise and predictable orthodontic programming) and to the introduction of viscoelastic polymers with excellent aesthetic and functional features. The material most often used today to make clear aligners is PET-G (Table 1), an acronym for a complex organic molecule, polyethylene terephthalate glycol copolyester. This material comes in round or square sheets, in various thicknesses. Depending on the orthodontic method involved, different thicknesses (0.55, 0.65, and 0.75 mm) can be used sequentially to obtain a greater tooth movement, or a single thickness (usually 0.75 mm) is used for smaller movements.

PET-G is classified as a highly-transparent amorphous polymer having visco-elastic properties, i.e., its characteristics are between those of elastic and viscous materials [9]. This material has been the object of numerous in vitro and in vivo studies because the quantity and quality of orthodontic movements depend on this material’s physical properties [4]. Various factors can influence the behavior and characteristics of PETG aligners, some of them modifiable, while others relate intrinsically to the manufacturing technique involved. One of the latter concerns the thermoforming process used to fashion the aligners: the sheet is heated and shaped on a resin model (under direct pressure or by vacuum forming) on which the required orthodontic movement has already been programmed. This process alters the polymer’s amorphous structure, increasing its elastic modulus, but also its capacity to absorb water [10].

The oral cavity–in which the aligners are immersed for up to 17–18 h a day (depending on the clinical recommendations for their use)–also has a dramatic effect on the properties of the material. This wet environment has a pH that varies between 6.5 and 7.4. Water absorption by the polymer alters its mechanical properties as well as its structure: the water absorbed seems to trigger a “hydrolytic degradation” phenomenon, disrupting the inter- and intra-chain hydrogen bonds, and modifying spatial volume between polymers chains [11]. It has been demonstrated that these materials only transmit from 42.29% to 66.56% of their initial force after being immersed for 3 h in water at 37 °C [9]. Bao et al., demonstrate that absorbed water acts as a plasticizer and enhances the relaxation process of the polymer matrix [12]; desorption and re-absorption experiments demonstrated that the structural relaxation is irreversible upon desorption, consistent with the plasticizing effect of absorbed water.

It is also worth noting that oral temperature can rise to as high as 57° from the ingestion of hot liquids, and remain high for several minutes before returning to normal. Stress-strain diagrams show a flattening of the curve, with a loss of elasticity, due to water molecules occupying space between polymer network chains. Parametric measurements obtained using laboratory mechanical tests point to a rapid decline in the force applied to the tooth [13].

On the other hand, the warmth of the oral cavity would seem to improve other properties of the polymer, such as Young’s modulus and its tensile stress. But warmth in a wet environment facilitates water absorption as well. Numerous studies have investigated the effects of temperature and repeated loading on aligners over time. An aligner submitted to 1000 thermal cycles (corresponding to 36.5 days of use in clinical practice) apparently becomes stiffer due to changes in its crystalline structure, but its deflection and the forces it exerts do not change. On the other hand, 100 loading cycles (inserting and removing the appliance) increase the aligner’s stiffness and reduce the force it exerts [14]. In fact, the deflection of a visco-elastic material under constant loads increases over time (a phenomenon known as creep), and at a constant deflection their load decreases (a phenomenon known as stress relaxation) [15].

A careful literature review reveals the extent of the impact of chemical and physical variables on the material used to make aligners. One aspect that tends to be overlooked, however, concerns the conditions in which aligners are stored when not in use (i.e., from 3 to 8 h a day, depending on the orthodontic technique involved). No information was found on how this variable affects the material. In other words, no studies were identified that examined the various options for storing and disinfecting aligners using different substances and compared the effects of different solutions on aligner deformation and weight change.

The purpose of the present study was to compare the various methods for storing aligners, assessing the in vitro consequences of appliance deformation and water absorption. Two research hypotheses were examined: the first was that storing aligners in a wet environment affected aligner deformation; the second was that a wet environment gave rise to a weight gain.

## 2. Materials and Methods

To establish which method for storing and disinfecting aligners causes the least structural changes (expansion or shrinkage), 50 aligners were prepared from sheets of Duran White Pd (Scheu Dental, Iserlohn, Germany), using the Clear Aligner method (Scheu Dental, Iserlhon, Germany). All aligners were fashioned on the same resin model obtained from a randomly-selected upper arch. This type of PETG sheet material, 0.75 mm thick, was chosen because it is the most frequently used size to make aligners according to various orthodontic methods. The white variant was chosen because it is easy for the intra-oral scanner to read for the purpose of dimensional changes. The PETG sheets all came from the same batch and had been stored according to the manufacturer’s instructions.

The Clear Aligner technique used incorporates a pressure molding machine (Biostar, Scheu Dental, Iserlohn, Germany) and involves heating the sheet to a preset temperature of about 200 °C (for 25 s), then shaping it under positive pressure (of approximately 6 atm) for 60 s. The whole process was completed by the same operator, who has considerable experience in the preparation of aligners. After cooling, the molded sheet was removed from the resin model, then cut and shaped to obtain the samples.

**Figure 1:**
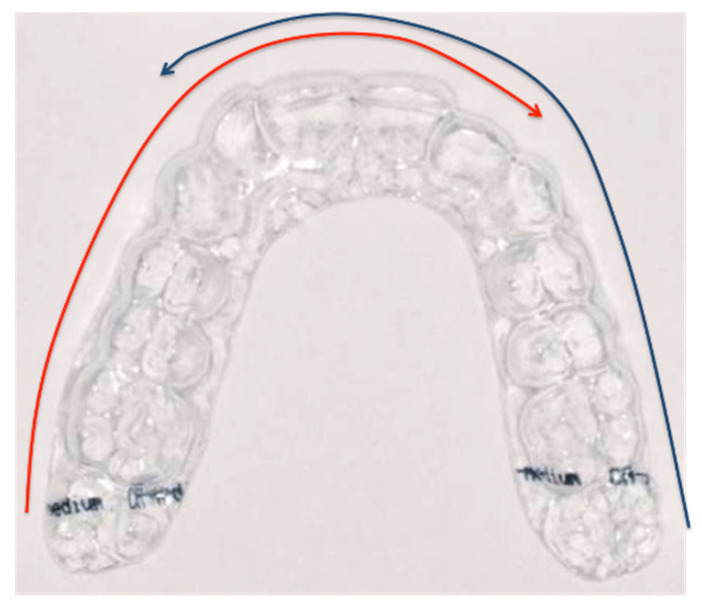
Path of the scanning technique.

At T0, the samples were numbered and weighed using precision scales (±0.01 g, DIPSE. TP-500), then optically scanned (CS 3600 Intra-Oral Scanner (IOS), Carestream Dental, Rochester, NY, USA), according to a precise scanning protocol. Three outer surfaces were considered for scanning purposes: the vestibular; the occlusal/incisor margin; and the lingual palatal tooth side The inner surface of the aligners was not scanned. The scanning technique (Figure 1) covered the teeth from the left lateral incisor to the right second molar, and from the right lateral incisor to the left second molar [16].

The data acquired were digitally recorded in a standard STL file format. The aligners were then randomly divided into two groups, one for simulating their storage in wet conditions, the other in dry conditions. A manual randomization procedure was used, based on the distribution of sequentially-numbered, opaque, sealed envelopes containing previously-prepared allocation cards.

At T1, immediately after their fabrication, the samples were submitted to various hydrothermal cycles for a period of two weeks. Group 1 consisted of 30 aligners stored for 18 hours a day in artificial saliva (grade 3 water, ISO3696) [17] in an incubator (Water Bath, Joanlab, BHS-1) at 37 °C, then for the remaining 8 h a day: 10 samples (Group 1A) were stored at room temperature in an aqueous solution obtained by dissolving an anhydrous citric acid and bicarbonate material specifically designed for cleaning aligners (Cetron, Scheu Dental GmbH); 10 samples (Group 1B) were stored at room temperature in a disinfecting solution of chlorhexidine digluconate (CURASPET ADS 0.20%); 10 samples (Group 1C) were stored at room temperature in distilled water; 20 samples (Group 2D) immersed in artificial saliva [18] (Table 2) in an incubator at 37° for 16 h a day, then stored in dry conditions at room temperature for the remaining 8 h (control group). Group 2D included 20 specimens in order to achieve a similar number of specimens between the wet groups and the dry group. Antimicrobial solutions were replaced before every new cycle.

At T2, all aligners in groups 1 and 2 were scanned again using the same tools and the same method as at T0. Wet specimens were dried using a dental air spray for 10 s immediately before scanning. Then the scans obtained at T2 were superimposed on those obtained at T0, and the aligners were weighed again. The two variables of interest for the purposes of this study were the aligners’ linear deformation (in millimeters) and weight (in grams). ICCs (Intraclass Correlation Coefficient) were used for reliability testing, with a target value of 0.8 and a 95% confidence interval (CI) of 0.2.

**Figure 2:**
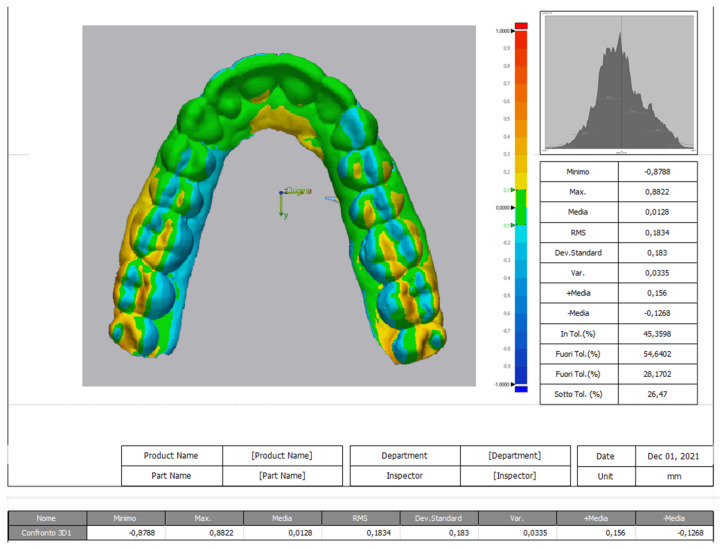
Superimposing the mesh with the Geomagic software.

The optical scan obtained dimensional differences between observation times (Geomagic Design X Control 3D System) used the value at T0 for reference and imported the scan obtained at T2. The software tools enabled a manual (mesh-based) alignment of the two objects (the scans of the aligners) to obtain the first result. Then, using 3D comparison software (a subprogram of Geomagic Design), the two 3D scans were superimposed (Figure 2). The resulting report provided the mean overlap and its standard deviation, in mm, for each aligner.

Each aligner’s linear deformation was calculated as the mean of the discrepancies found on superimposing the two scans obtained at T0 and T2. The software generated two types of data: (1) a central indicator; and (2) the deformation variability, or the standard deviation of the deformations at T0 and T2, corresponding to the differences in expansion or contraction between the different points recorded by the system. Weight differences were calculated as the difference between the weights recorded at T0 and T2. Dimensional change and weight change were expressed as medians and interquartile ranges (IQR), and graphically presented using box and whisker plots. The dimensional changes in Groups 1A, 1B, 1C, and 2D were compared using the nonparametric Kruskal-Wallis test and the Mann-Whitney test. Given the exploratory nature of the study (hypothesis generator), no correction for multiple tests was applied (between dimensional change and weight change). All tests were two-tailed and used a pre-set alpha of 0.05. Statistical analyses were conducted using software (R 4.0, R Foundation for Statistical Computing, Vienna, Austria).

## 3. Results

The linear deformations among the various groups of aligners, the median deformation, and the deformation variability were considered. Descriptive statistics for these findings are presented in Table 3.

**Figure 3:**
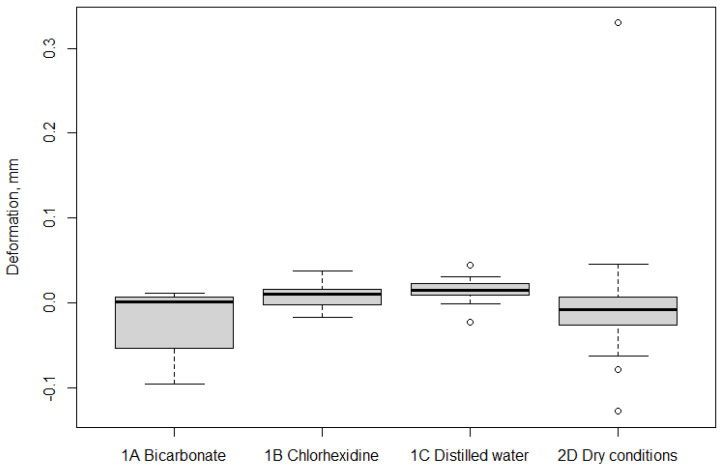
Deformation data profiles of the four groups: bicarbonate (1A), chlorhexidine (1B), distilled water (1C), and dry conditions (2D). Box and whiskers plot.

The median deformation amounted to: Group 1A 0.001 (IQR −0.041 to 0.006); 0.01 (IQR −0.001 to 0.0159) in group 1B; 0.015 (IQR 0.009 to 0.021) in group 1C; and −0.008 (IQR −0.023 to 0.005) in group 2D (Figure 3). As for deformation variability, the median values were: 0.068 (IQR 0.54 to 0.088) in group 1A; 0.087 (IQR 0.064 to 0.121) in group 1B; 0.091 (IQR 0.072 to 0.131) in group 1C; and 0.157 (IQR 0.096 to 0.212) in group 2D. Median deformation and deformation variability showed the minimum value (0.001mm, 0.068mm) in group 1A and the maximum value (0.091 mm, 0.157 mm) in group 2D. A high number of data outliners in the bar and whisker graphs for Groups 1C (2 outliers) and 2D (3 outliers) were noted.

### 3.1. Group Comparison

#### 3.1.1. Among Only Wet-Stored Groups

There was no statistically significant difference between groups 1A and 1B in terms of deformation (*p* = 0.08) or deformation variability (*p* = 0.35). (Upper portion of Table 4) Likewise, no statistically significant difference was noted between Groups 1B and 1C in either deformation (*p* = 0.43) or deformation variability (*p* = 0.68). On the other hand, the data indicated a statistically significant difference between groups 1A and 1C in terms of deformation (*p* = 0.006*), but not their variability (*p* = 0.19) (Table 4).

#### 3.1.2. Among Dry-Stored and Wet-Stored Groups

In a second analysis, group 2D was compared with groups 1A, 1B, and 1C (Lower portion of Table 4). It emerged that the linear deformation was significantly greater in group 1C than in group 2D (*p* = 0.02*), while it was similar between groups 1A and 2D (*p* = 0.81), and the difference between groups 1B and 2D only neared statistical significance (*p* = 0.07). Deformation variability was significantly greater in group 2D than in groups 1A (*p* = 0.01*) or 1B (*p* = 0.01*), while the difference between groups 1C and 2D only neared statistical significance (*p* = 0.07).

### 3.2. Weight Difference

The descriptive statistics of the aligners’ weight differences are shown in Table 5.

**Figure 4:**
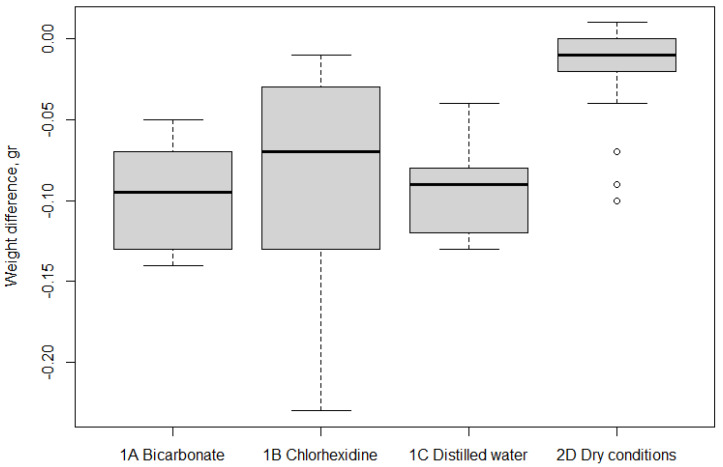
Weight difference in the four groups: bicarbonate (1A), chlorhexidine (1B), distilled water (1C), and dry conditions (2D). Box and whiskers plot.

An increase in weight from T0 to T2 in all the groups was noted, which ranged from −0.095 to −0.010 gr, with the greatest weight gain occurring in Group 1A (−0.095 g) and the smallest in Group 2D (−0.010 g). The group comparisons (Table 4) identified no statistically significant difference between the weight gains in Groups 1A and 1B (*p* = 0.54), 1B and 1C (*p* = 0.51), or 1A and 1C (*p* = 0.99). When Group 2D was compared with the other groups, the weight gain was more limited in Group 2D than in Groups 1A (*p* = 0.0001*), 1B (*p* = 0.0002*) or 1C (*p* = 0.0002*). The graph of weight differences (Figure 4) shows that the median weight gain in Group 2D corresponds to the maximum value recorded in Group 1B. This latter group’s weight gain was more variable than that of the other groups. A high number of data outliners in the bar and whisker graphs was noted for Group 2D.

## 4. Discussion

With respect to the first research hypothesis regarding the median deformation, aligners expanded to some degree in all three groups stored in wet conditions (the difference ranging between +0.01 and +0.015 mm), while there was a shrinkage (−0.08 mm) in the group stored in dry conditions, as expected. These findings are compatible with previous reports [9,10,11,12] of water being able to penetrate the polymer structure of the PETG during the thermal cycle, interacting with its intermolecular bonds. It is worth noting, on the other hand, the shrinkage occurring in the material alternately exposed to the moisture of the oral cavity and then to dry storage conditions (Group 2D) was probably due to the previously-absorbed water subsequently evaporating. The first research hypothesis was upheld.

The findings regarding deformation variability express the distribution of the deformation over the surface of the aligners. This amount of variation was similar among the three groups of aligners stored in wet conditions, demonstrating a degree of homogeneity in their deformation. There was a greater deformation variability in group 2D, however (0.157): aligners stored in dry conditions when not “worn.” This finding suggests that the cycles of water absorption and evaporation deform some areas of the aligner’s surface more than others.

Comparing the groups of aligners stored in wet conditions (1A, 1B, 1C), the expansion was particularly significant in the group kept in distilled water, especially in comparison with aligners stored in a bicarbonate solution, while there was no significant difference between the groups stored in bicarbonate solution versus chlorhexidine. The group of aligners stored in a dry condition shrank in volume to a significant degree more when compared with those stored in distilled water, and to a lesser degree than those stored in a bicarbonate solution, (with a difference nearing statistical significance), and even less compared with those stored in chlorhexidine. When evaluating deformation variability similar trends were noted: the difference between Group 2D and Groups 1A and 1B proved statistically significant (*p* = 0.01 *), while it was only weakly significant in relation to Group 1C (*p* = 0.07). In summary, distilled water seemed to have a more variable expansive effect, while aligner expansion and its variability were more limited in the other two “wet” conditions, and storage in dry conditions made the PETG shrink slightly.

With respect to the second research hypothesis, an increase in weight of all aligners was seen, so they all absorbed water to some degree; this finding is comparable with previous studies on the issue [11]. Weight gain was greatest in Group 1A (−0.095 g) and Group 1C (−0.090 g), followed by Group 1B (−0.070 g). Group 2D, cyclically switching between wet and dry conditions, also gained weight (−0.010 g), but significantly less than aligners stored in wet conditions (*p* = 0.0001*, *p* = 0.0002*, *p* = 0.0002*). It is worth emphasizing that the small weight gain of Group 2D occurred despite shrinkage in aligner volume, suggesting that hydrothermal cycles of water absorption and evaporation could promote a slight contraction of the material despite an increase in its weight. Also, the second research hypothesis was upheld.

Judging from the data in the present study, storing aligners in dry conditions at home when not worn seems to cause less surface deformation (swelling) by allowing absorbed water from the oral cavity to evaporate, indicating a slight shrinkage. In this study, it was assumed that the dimensional changes of the outside surface influence forces applied to the tooth surface: if the outer surface expands, it seems logical for the inner surface to do the same; and, vice versa, if the outer surface shrinks, so will the inner surface. In clinical terms, in the case of shrinkage, this could mean a better fit for the aligner when it is put back in the oral cavity, a factor that could combat the known problem of ‘stress relaxation’ [15] caused by the repeated insertion and removal of the aligner. An expansion could exacerbate the stress relaxation phenomenon. In fact, patients very often report a worsening fit and reduction in the perceived tooth force exerted only within days of receiving a new aligner.

The present study shows that the type of solution used when not wearing the aligner can make a difference when aligners are stored in wet conditions. While there was no statistically significant difference between the effects of the disinfectants usually chosen for aligners (chlorhexidine or a citric acid and bicarbonate solution), using distilled water resulted in greater deformation in the PETG material.

Given these findings, it seems advisable to store aligners in dry conditions most of the time they are not in use and place them only briefly in disinfectant solutions (avoiding the use of distilled water). On the microbiological level, the most effective solution is chlorhexidine, which is capable of binding to the bacterial membrane and has a broad spectrum of action against Gram-negative and -positive bacteria [19]. In the present study, its use did not alter the color or stain the aligner, probably because PETG is a polished, non-porous polymer (unlike dental enamel), so the biguanide molecules in chlorhexidine were unable to penetrate the material, however smaller water molecules could.

Current advancements and research on aligners include the use of multi-layer materials to overcome the drawbacks of a single composition material. Given the role of a wet environment in the alteration of polymers, it might be worth adding a thin waterproof outer layer to preserve an inner core against hydrothermal aging.

The main limitation of the present study lies in that it was conducted as an in vitro experiment. Another weakness concerns the fact that the inside of the aligner was not scanned. Although the deformation occurring on the outside presumably coincided with the same behavior on the inside, this aspect was not specifically tested.

## 5. Conclusions

Within the limitations of this in-vitro investigation, the following conclusions may be drawn.

1. Storing aligners in distilled water when they were not in use caused the most linear deformation (expansion) of the PETG, and showed the greatest deformation variability, while two solutions (a recommended cleaning solution or a disinfectant) both performed better. Storing aligners in dry conditions led instead to a slight shrinkage of the material, with a lesser degree of deformation variability, and
2. In all the cases tested, there was evidence of aligner weight gain, which was highest when stored in distilled water and lowest when stored in dry conditions while not being “worn”.

## Figures and Tables

**Table 1:** PET-G Datasheet.

PET-G Datasheet	Guideline	Value
Designation	-	Polyethylene terephthalate glycol-modified
CAS-Number	-	25640-14-6
Form	-	Solid
Color	-	Transparent
Density	ISO 1183	1.27 g/cm^3^
Water absorption (24 h at 23 °C)	ISO 62-4	0.2%
Tensile strength	ISO 527	53 MPa
E-modulus	ISO 527	2200 MPa

**Table 2:** Composition of artificial saliva (pH = 6.5).

Compound	Content (g/L)
NaCl	0.6
KCl	0.72
CaCl_2_⋅2H_2_O	0.22
KH_2_PO_4_	0.68
Na_2_HPO_4_⋅12H_2_O	0.856
KSCN	0.06
NaHCO_3_	1.5
C_6_H_8_O_7_	0.03

**Table 3:** Median deformation and deformation variability (mm).

Group	Median Deformation (IQR)	Deformation Variability (IQR)
1A (Bicarbonate)	0.001 (−0.041; 0.006)	0.068 (0.054; 0.088)
1B (Chlorhexidine)	0.010 (−0.001; 0.0159)	0.087 (0.064; 0.121)
1C (Distilled water)	0.015 (0.009; 0.021)	0.091 (0.072; 0.131)
2D (Dry)	0.091 (0.072; 0.131)	0.157 (0.096; 0.212)

**Table 4:** Pair-wise group comparison: Deformation, Deformation Variability, and Weight.

Comparison	*p* Value (Deformation)	*p* Value (Variability)	*p* Value (Weight)
1A vs. 1B	0.08	0.35	0.54
1B vs. 1C	0.43	0.68	0.51
1C vs. 1A	0.006 *	0.19	0.99
2D vs. 1A	0.81	0.01 *	0.0001 *
2D vs. 1B	0.07	0.01 *	0.0002 *
2D vs. 1C	0.02 *	0.07	0.0002 *

* Indicates a statistically significant difference (*p* < 0.05).

**Table 5:** Weight difference (gr).

Group	Weight Difference (IQR) T0-T1
1A (Bicarbonate)	−0.095 (−0.125; −0.070)
1B (Chlorhexidine)	−0.070 (−0.125; −0.037)
1C (Distilled water)	−0.090 (−0.112; −0.082)
2D (Dry)	−0.010 (−0.020; 0.000)

## Data Availability

Not applicable.
